# Three-Dimensional Structure of the Human Myeloma IgG2

**DOI:** 10.1371/journal.pone.0064076

**Published:** 2013-06-07

**Authors:** Sergey Ryazantsev, Vladimir Tischenko, Christopher Nguyen, Vyacheslav Abramov, Vladimir Zav'yalov

**Affiliations:** 1 Department of Biological Chemistry, David Geffen School of Medicine at University of California Los Angeles, Los Angeles, California, United States of America; 2 Institute of Theoretical and Experimental Biophysics, Russian Academy of Sciences, Pushchino, Russia; 3 Institute of Immunological Engineering, Lyubuchany, Russia; 4 Joint Biotechnology Laboratory, Department of Chemistry, Mathematics and Natural Sciences Faculty, University of Turku, Turku, Finland; California State University Fullerton, United States of America

## Abstract

Human immunoglobulin G, subclass 2 (hIgG2), plays an important role in immunity to bacterial pathogens and in numerous pathological conditions. However, there is a lack of information regarding the three-dimensional (3D) structure of the hIgG2 molecule. We used electron microscopy (EM), differential scanning microcalorimetry (DSC) and fluorescence for structural analysis of the hIgG2. DSC and fluorescence indicated two types of interaction between C_H_1 domain of Fab (antigen-binding fragment/subunit) and C_H_2 domain of Fc (complement fixation fragment/subunit) simultaneously present in the sample: close interaction, which increases the thermostability of both, C_H_1 and C_H_2 domains, and weak (or no) interaction, which is typical for most IgGs but not hIgG2. Thermodynamics could not determine if both types of interactions are present within a single molecule. To address this question, EM was used. We employed a single-particle reconstruction and negative staining approach to reveal the three-dimensional structure of the hIgG2. A three-dimensional model of hIgG2 was created at 1.78 nm resolution. The hIgG2 is asymmetrical: one Fab subunit is in close proximity to the upper portion of the Fc subunit (C_H_2 domain) and the other Fab is distant from Fc. The plane of Fab subunits is nearly perpendicular to Fc. EM structure of the hIgG2 is in good agreement with thermodynamic data: a Fab distant from Fc should exhibit a lower melting temperature while a Fab interacting with Fc should exhibit a higher melting temperature. Both types of Fab subunits exist within one molecule resembling an A/B hIgG2 isoform introduced earlier on physicochemical level by Dillon et al. (2008). In such an arrangement, the access to the upper portion of Fc subunit is partially blocked by a Fab subunit. That might explain for instance why hIgG2 mildly activates complement and binds poorly to Fc receptors. Understanding of the three-dimensional structure of the hIgG2 should lead to better design of antibody-based therapeutics.

## Introduction

The immunoglobulin G (IgG) molecule is composed of two Fab subunits that are linked to Fc subunit via a hinge region. Fab is responsible for antigen recognition and binding. Fc is responsible for effector functions such as classical complement cascade activation triggered by C1q (first component of complement) binding, macrophage activation triggered by interaction of immune complexes with Fc receptors, etc. Human IgG (hIgG) subclasses exhibit a tremendous variety of functions while in general, the structure of Fab and Fc is quite conservative. Different hIgG subclasses have different abilities to activate the classic complement cascade, which are mediated by the structural properties of the hinge region, and/or by the structure of the C1q binding site located in C_H_2 domains [1]–[4]. A strong modulating effect of the lower hinge region of hIgG1 on C1q binding also may be mediated via a change in the predominant shape of an hIgG1 molecule [5], i.e. the binding site may be opened or closed for ligand binding according to the reciprocal arrangement of Fab and Fc subunits.

Indeed, it was found by electron microscopy that about 70% of molecules of intact myeloma hIgG1 were not planar but had a tripod-like shape and were flexible within this conformation [6]. This hIgG1 sample demonstrated partial complement-activating ability due to its predominant tripod-like shape and flexibility, which together make the C1q-binding site more available for docking. An intermediate level of C1q binding activity for hIgG1 is due to its flexibility when the C1q-binding site is available only part of the time. In contrast, the truncated hIgG1 myeloma proteins Dob and Mcg [7] exhibit the absence of the C1q-binding ability and complement dependent cytotoxity. This is attributed to the lack of the hinge region, which leads to the rigid T-shaped structure, which obstructs the docking of C1q.

The pig non-precipitating anti-dinitrophenyl IgG antibodies are very rigid tripod-like molecules with minimal flexibility [8]. This subtype of IgG exhibits a high level of complement-binding activity. Its activity is higher than, for instance, hIgG1 [6], due to the rigid tripod-like shape, apparently favorable for C1q binding [8].

hIgG2 mildly activates complement and binds poorly to Fc receptors. The mild ability of hIgG2 to bind C1q may be explained by the significant differences in the sequence and structure of the lower hinge in comparison with hIgG1, which in turn may mediate a predominant shape of hIgG2 molecule unfavorable for binding. It is also known that hIgG2 has the most rigid structure of all hIgG subclasses [9], [10]. Another interesting feature of hIgG2 is the formation of isoforms when disulfide bonds are arranged in various fashions [11]–[14]. There are three isoforms currently known for hIgG2 [11]–[14]: **A** – canonical IgG intersubunit arrangement of disulfide bonds when both Fab's are connected to Fc via the hinge region; **B** – when both Fab's are connected to C_H_2 domain of Fc directly, using unusual disulfide bonds formed at the interface between variable (V) and constant (C) parts of Fab; **A/B** – asymmetric Fab arrangement within the IgG molecule, when one Fab is connected in **A**-fashion and another in **B**-fashion. In this article, we sometimes use the term “conformer” to emphasize isoforms' structural differences. A majority of intact hIgG2 molecules are present in A/B isoform [11]–[14]. Liu et al. [12] studied the maturation of hIgG2 in the bloodstream and found that during the maturation process, hIgG2 was initially present in the **A**-form, then quickly turned into the **A/B**-form and thereafter – slowly into the **B**-form. A similar phenomenon was observed *in vitro* during storage of the sample [12].

Lightle et al. (2010) [15] have designed a series of cysteine to serine mutations on a common IgG2 antibody backbone to examine the difference of the IgG2 disulfide isomers in effector functions. They observed structural homogeneity with these mutants and mapped the locations of their disulfide bonds. However, these mutations have little to no effect on FcγR (Fc gamma receptor) or C1q binding, indicating that isomer composition has no impact on the accessibility of the binding sites.

Despite a significant role of hIgG2 antibodies (about 25% of total hIgG in serum [16]) in adaptive and innate immunity and its involvement in various pathologies [17]–[19], there is no detailed data on the structure of the intact hIgG2. The role of the different isoforms in immune response and pathological conditions is also unclear.

Investigation of the hIgG2 structural properties was a continuation of our efforts to unveil structure-function relationships in different subclasses of human IgG [6], [8], which we performed in 1990. Thermodynamic analysis showed unusual melting properties for hIgG2, which were difficult to interpret at that time. EM was inconclusive showing “strange” butterfly shapes, which, again, were difficult to interpret. The project was abandoned for many years. Only a small number of glass plates with EM micrographs of hIgG2 survived. They were revisited in recent years when modern EM techniques became available. It started as a small project for CN to practice single particle 3D reconstruction, but the first 3D data on hIgG2 was so interesting that the project was expanded to include all previously collected data. Since initial data collection was performed many years ago, we were limited only to the available data. This paper contains two quite independent parts – EM and thermodynamic studies. We believe that this paper would not have been possible without the thermodynamic part – it first demonstrated the unusual thermal properties of hIgG2 and prompted further investigation on the EM level. Preliminary 3D model of hIgG2 was difficult to interpret. At some point, we learned about hIgG2 isoforms [11]–[14] and soon discovered that our 3D model is in good agreement with **A/B** hIgG2 isoform.

In our opinion, this work shows the power of a combined approach – thermodynamics raised the questions and EM, with very limited resources, helped to explain the observed phenomenon.

## Results and Discussion

### Thermodynamic and fluorescent studies

Differential Scanning Microcalorimetry (DSC) has been successfully used to study the differences between hIgG subclasses and to test their conformation [20]–[22]. At acidic pH values, all IgG and isolated Fc fragments studied exhibit a low-temperature peak of heat absorption, which corresponds to melting of the C_H_2 domains [20]–[25]. Thermostability of C_H_2 domains is lower than that of other domains because interactions in the pair of C_H_2 domains are weaker in comparison with the pairs V_L_-V_H_, C_L_-C_H_1 and C_H_3- C_H_3 domains [7], [26]–[28]. Fab subunits and C_H_3 domains are normally melted in a high-temperature area [20]–[25].

**Figure 1:**
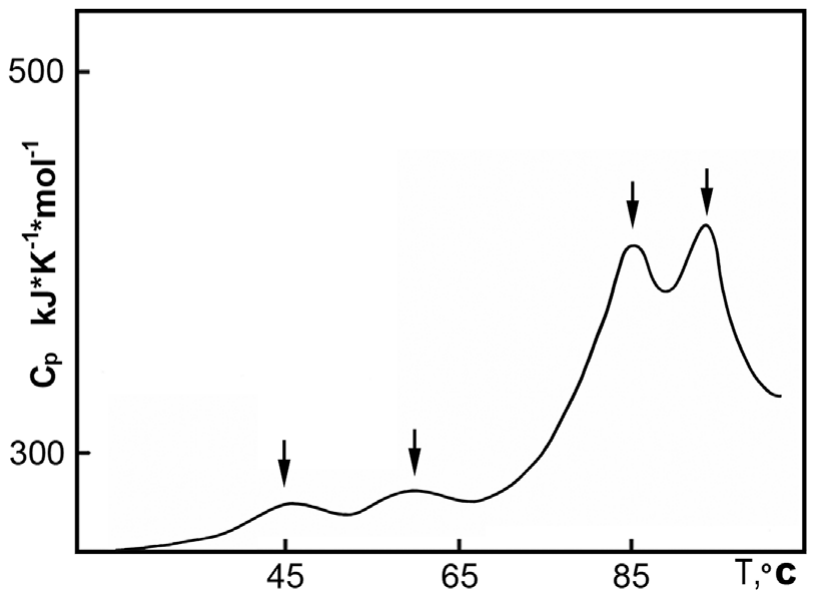
Temperature dependency of the molar partial heat capacity of the hIgG2 in 10 mM phosphate buffer, pH 6.0. Peaks of the heat absorption at 45, 58, 85 and 94°C are indicated by arrows.

Figure 1 shows dependency of molar partial heat capacity on the temperature for hIgG2 in 10 mM phosphate buffer, pH 6.0. In the low-temperature area of the DSC thermogram two peaks of 45 and 58°C are observed. The first peak is typical, representing the melting of the C_H_2 domain(s) and is observed on the melting curves of all IgGs previously studied [20]–[25]. The second peak is unique and is observed only in hIgG2 samples stored for some period of time. In high-temperature area, two peaks of 85 and 94°C are presented. Interpretation of these peaks requires additional studies. Therefore, a special approach was developed [29]. In this approach, specific domain(s) of the IgG molecule were selectively labeled with a fluorescent label, making it possible to analyze the conformational changes in the domain(s) during the melting process. For details, refer to Tischenko et al. (1998) [29]. We implemented this approach to study the hIgG2 sample.

**Figure 2:**
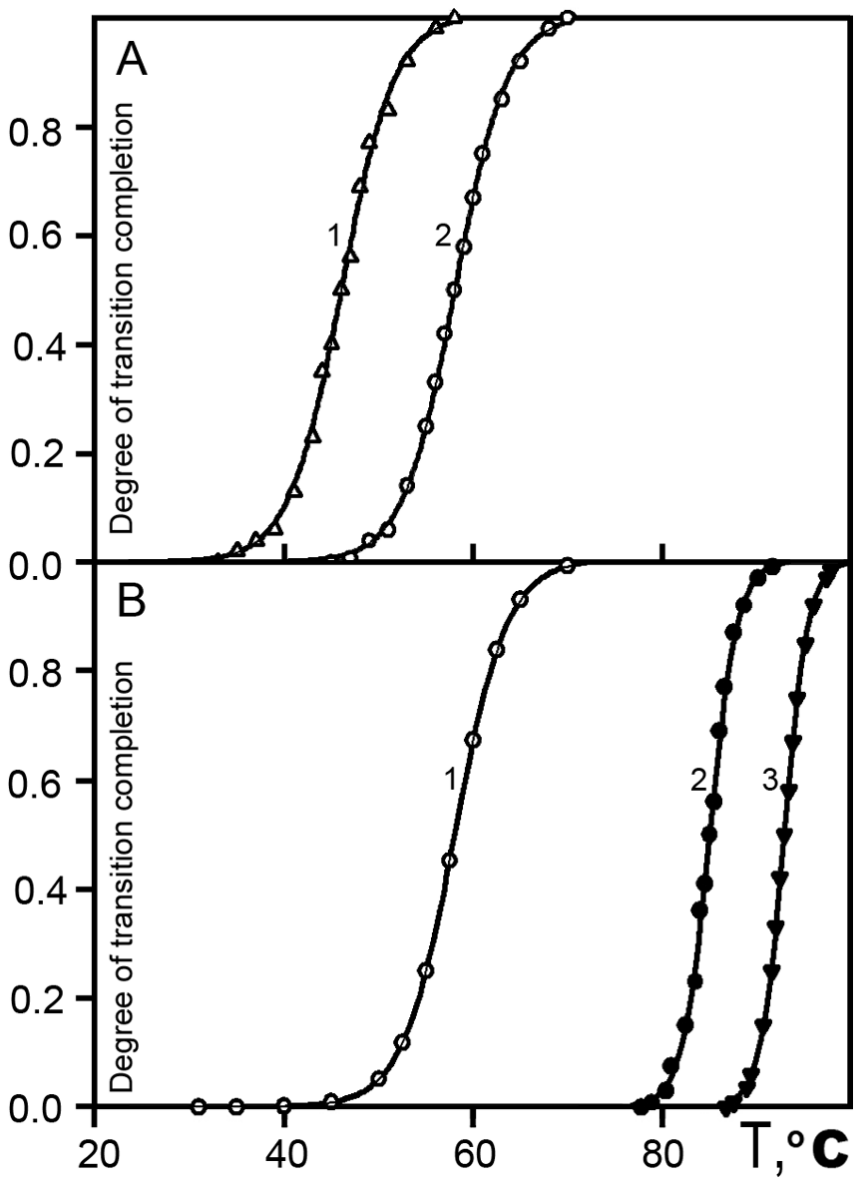
Temperature-dependent changes of the fraction of the denatured C_H_1 and C_H_2 domains of the hIgG2 determined with a fluorescent label in 10 mM phosphate buffer, pH 6.0. (A) FITC-labeled C_H_2 domain(s). The midpoint of the curve 1 and 2 is 45°C and 58°C, respectively. (B) FITC – labeled C_H_1 domain(s). Midpoint of curve 1 is 45°C; curve 2, 85°C, and curve 3, 93°C.

Figure 2 shows the melting curves for FITC-labeled (fluorescein isothiocyanate) C_H_2 and C_H_1 domains. The melting process of C_H_2 domains is described by two curves. Figure 2A curve 1 corresponds to the peak with Tm  = 45°C on the DSC thermogram (Fig. 1), and Figure 2A curve 2 corresponds to the peak with Tm  = 58°C. These data indicate that both low-temperature peaks on the DSC thermogram (Fig. 1) represent the melting of the C_H_2 domains. Usually, only one low-temperature peak corresponds to the melting of the C_H_2 domain in IgG [20]–[25]. The presence of two low-temperature peaks corresponding to the melting of C_H_2 domains was not previously reported.

Similarly, we investigated the temperature transition in the FITC-labeled C_H_1 domain. Figure 2B shows that melting of the C_H_1 domain(s) is represented by two curves corresponding to two high-temperature peaks on the DSC thermogram with Tm equal to 85 and 94°C (Fig. 1). These data show that melting of the C_H_1 domains in the hIgG2 molecule contributes to both high-temperature peaks. Thus, based on two independent methods, we could conclude that C_H_2 and C_H_1 domains in hIgG2 existed in two distinguishable states with different thermostability. However, thermodynamic data were not conclusive regarding the nature of these conformers; it cannot distinguish between a mixture of A and B and hybrid molecules A/B. This distinction requires more direct methods of structural analysis such as EM and 3D reconstruction.

### Electron Microscopy and 3D reconstruction

Determination of the hIgG2 3D structure was provided by single particle reconstruction with EMAN [30]. Details of the procedure are described in “Material and Methods.” The single particle reconstruction approach is based on the assumption that the sample is homogeneous and that particles are randomly oriented on an EM grid [31], generating different projections. These projections may be combined to create a 3D model [32]. As mentioned above, flexibility is an important feature of many IgG molecules and modulates many functions of the IgG. In general, flexibility of the molecule makes single-particle 3D reconstruction difficult if not impossible. However, hIgG2 is most rigid between human IgG subclasses [9], which makes 3D reconstruction feasible.

3D reconstruction was performed using 3900 selected ‘good’ particles. Details of 3D reconstruction are described in the correspondent section of the “Supplemental Information” and summarized in Fig. S1.

Due to concerns regarding reliability of 3D reconstruction on small datasets with negatively stained samples we performed vigorous tests on our model:

- We tested four initial models – 5 random blobs of different sizes, ellipsoid with 1∶2 axis ratio, ellipsoid with 1∶3 axis ratio, and sphere. In all cases, we obtained very similar models (data not shown), which indicated that there was no “model bias” in our reconstructions.
- We analyzed model's convergence – model converged completely (data not shown).
- We analyzed the correlation between corresponding projections of the model, class averages and individual raw particles (Fig. S1B), and found them to be in remarkable agreement.
- We analyzed the Euler space filling (Fig. S1C), and found that the classes were uniformly spread in the space, which meant the particles were randomly oriented on the support film.
- We tested the resolution of the model using *eotest* (Fig. S1D) in EMAN – the resolution based on Fourier Shell Correlation, FSC  = 0.5 criterion [33], [34] was 1.78 nm.

**Figure 3:**
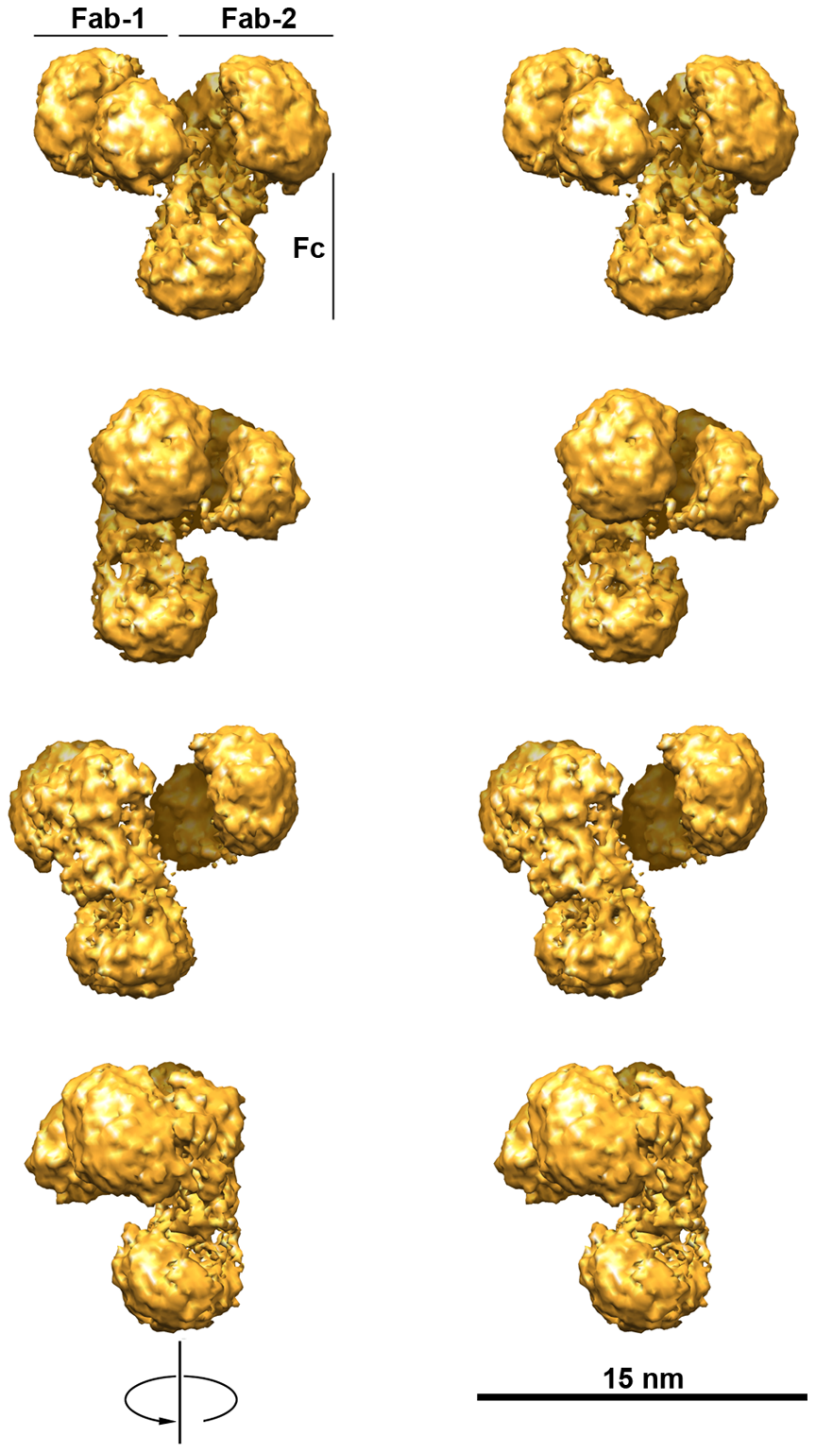
Stereo-view of hIgG2 3D model at 1.78 nm resolution. From top to bottom, the model is rotated 90° clockwise along the vertical axis of Fc. The model is oriented so that Fab subunits are on top. Fab-1 (see text for details) is at top left. The density threshold cutoff determined in EMAN is based on a molecular mass of 160 kDa and a protein density of 1.35 g/cm^3^. Weak electron densities, which connect parts of the molecule, are not visible at this threshold.

In the final 3D reconstruction we used a featureless Gaussian ellipsoid with 1∶2 axes ratio as the initial model. A model converged completely after 40 refine cycles with no symmetry implemented. Based on all our tests, we concluded that our model was truthful and properly represented the data. The stereo-view of obtained hIgG2 model is shown in Figure 3 at 1.78 nm resolution by FSC = 0.5 criterion.

To aid in the interpretation of the model, we used the visualization program, Chimera [35], to – through comparison to known structures – identify parts of the molecule, namely Fab and Fc subunits [7], [27], [28], [36]–[38]. Knowledge of the general architecture of the IgG molecule and relationships between its parts also helped to identify the parts of the hIgG2 molecule. We varied the density threshold cutoff (which determined where to lay isosurface) to trace connections between parts of the molecule. We also used segmentation [39] to outline the individual electron densities within the molecule. Our findings are summarized in Figures S2 and S3 in “Supplemental Information.”

The Fab subunit, which was distant from other parts of the hIgG2 molecule, was identified first by its typical appearance; we called it Fab-1 (Fig. S2A, B). A “connector” links Fab-1 to the rest of the molecule as is shown in Figure S3A, B. Since it is known that Fab connects to C_H_2, we assumed that the density connected to Fab-1 via a “connector” is a C_H_2 domain of Fc subunit. Once this was established, the part of the Fab- 1 closest to C_H_2 domain should be a *constant* (C) part, which contains C-domains. Another part of Fab-1, which is most distant from Fc is a *variable* (V) part of Fab-1. The structure of the C- and V- domains is shown in Figure S2A, B. On the cross-section of the hIgG2 model shown in Fig. S3B, there is an additional mass on top of C_H_2 domain, which is not continuous with the Fc subunit. This mass has been identified as a second Fab (thus, Fab-2) by similarity to Fab-1. Fab-2 has the same dimensions and structure as Fab-1. We assume that the part of Fab-2 closest to Fc is a C-part of Fab-2. Thus, Fab-2 is tightly attached to the upper part of Fc – the interface between Fc and Fab-2 is quite large; it includes not only C-part but also the area between V- and C-parts. The interface between Fab-2 and Fc is colored in magenta in Fig. S2D.

The Fc subunit is identified as part of the IgG molecule to which both Fabs are attached. Figure S2C, D shows the structure of the hIgG2 Fc subunit. The Fc subunit has a teardrop shape. Such shape of the Fc subunit in negatively stained samples of other IgGs was previously observed [6], [8], [40]. We interpret this as a possible effect of uranyl acetate staining on carbohydrates that fill the center of the Fc. There is no data available on aglycosylated Fc subunit structure. The gap between C_H_2 and C_H_3 portions of the Fc subunit on the model (Fig. S2C, D) is less pronounced than in the corresponding X-ray structure [28] perhaps due to the presence of stained carbohydrates. In the EM Fc model (Fig. S2C, D), the arrangement and shape of C_H_3 domains appear similar to the X-ray structure, while those of C_H_2 domains do not. In the side view (Fig. S2C, D), it is noticeable that the plane of C_H_2 domains is tilted substantially to the plane of C_H_3 domains – making the Fc subunit bend along the long axis. Note that this bend is visible only in the side view. The interface between Fab-2 and Fc subunits (magenta in Fig. S2D) is large, indicating tight interaction between two subunits.

### 3D structure of the hIgG2 – summary

**Figure 4:**
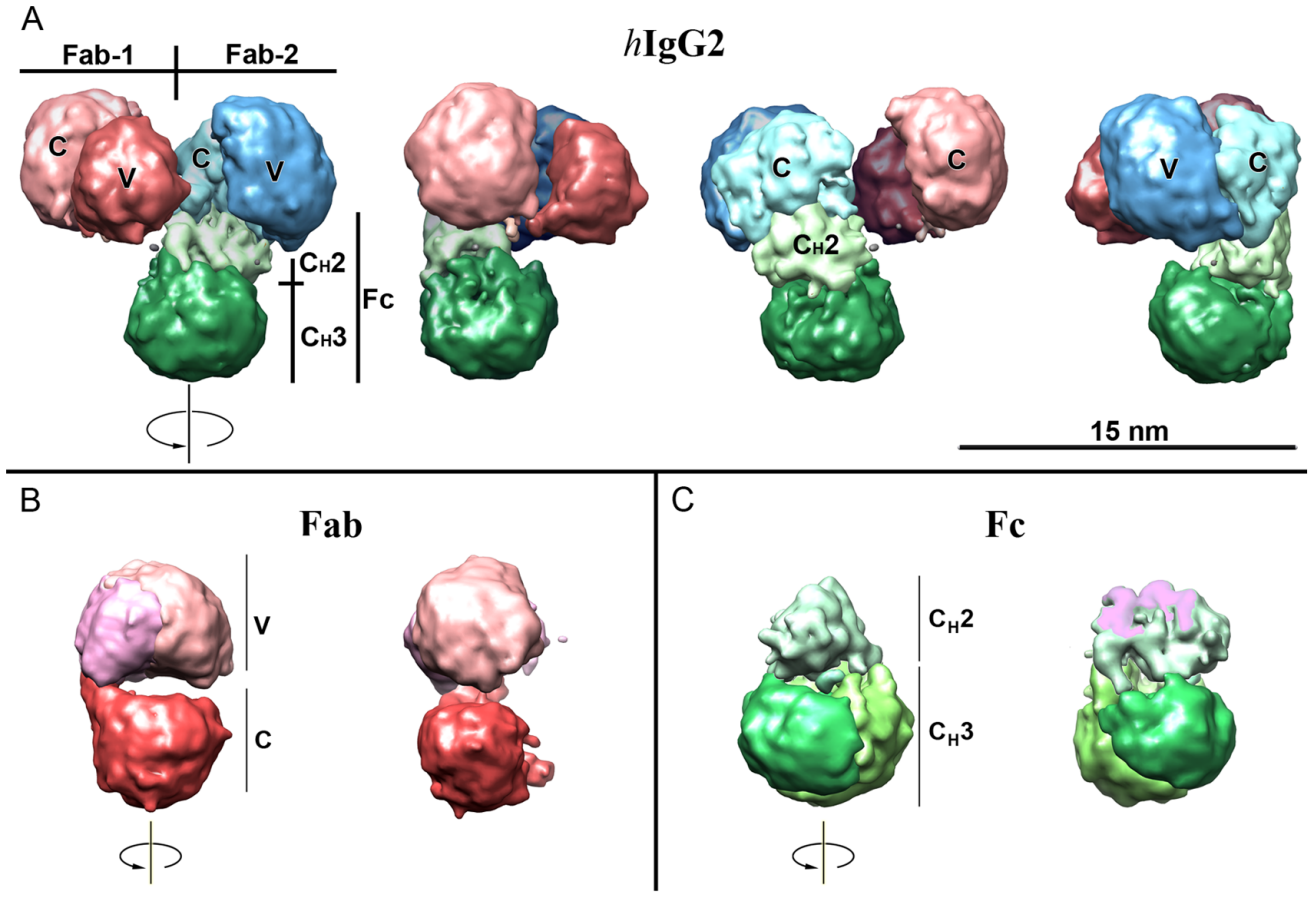
Human IgG2 3D model in detail. (A) The whole hIgG2 molecule; (B) Fab subunit; (C) Fc subunit. The model was segmented and visualized in Chimera. Each subunit is shown in a different color (green Fc; red Fab-1; blue Fab-2). Different segments within a subunit have different shades of the same color. From left to right, each model is rotated 90° clockwise along the vertical axis. In (C), the interface between C_H_2 and Fab-2 is colored magenta.

Figure 4 summarizes our findings regarding the hIgG2 3D structure. The model was segmented [39] in Chimera [35]. Segmentation permits display of the individual densities within the molecule. In our case, most of the functional parts of the hIgG2 molecule were segmented into separate volumes corresponded to structural elements of the IgG molecule: variable and constant parts of Fab subunit(s), C_H_2 part (two domains together) and C_H_3 domains (separately). To better understand the relationships between the subunits, a different color was assigned to each subunit. Within the subunit, each segmented part was colored in a different shade of the same color. Additional details may be seen in Figure 4B, C showing Fab and Fc subunits. Stereo-view of the hIgG2 molecule (Fig. 3) aids in seeing the relationships between subunits in 3D space.

Fab subunits of hIgG2 (Fig. 4B) have a typical appearance similar to those already published for other IgGs [27], [36]–[38]. Individual V- and C-parts of Fab are remarkably similar to published X-ray structures [26]–[38]. Fc subunit of the hIgG2 (Fig. 4C) is different from X-ray structures [26], [27] mainly in C_H_2 part. Within the hIgG2 molecule (Fig. 4A), subunits are arranged so that one Fab is in direct contact with C_H_2 domain(s) of Fc and another is free. Such conformation resembled the A/B isoform of hIgG2 known from physicochemical study [11]–[14]. Interestingly, the asymmetrical orientation of the Fab subunits within the IgG molecule was previously observed for IgG1 in crystal form [27]. The difference between those in crystal form and A/B isoform of hIgG2 [11]–[14] is that crystallized IgGs had an identical arrangement of disulphide bonds for both Fabs within the molecule while A/B isoform of hIgG2 has an entirely different arrangement of disulphide bonds for two Fabs. The asymmetrical shape of the hIgG1 and others IgGs is mainly a result of hinge flexibility while hIgG2 A/B isoform has a structural basis for asymmetry – different arrangement of disulphide bonds for each Fab within the molecule. In our model, the long axis of Fc is bent nearly 90° (Fig. 4C). Thus, Fabs are attached to Fc at an angle creating a tripod-like shape. The tripod-like shape for pig non-precipitating antibodies and hIgG1 was introduced earlier [6], [8]. It looks like the tripod-like shape of IgGs is more common than one would think: interestingly, the conformation of X-ray structure of the full-size IgG1 also resembled a tripod-like shape [27]. Acceptance of the idea that the functional properties of the IgG depend upon flexibility **and** predominant shape (including a tripod-like shape) may lead to more thoughtful design of antibody therapeutics.

### Computational analysis of the hIgG2 model

Proportions of the A, B, and A/B conformers (isoforms) in the hIgG2 sample may vary. Each conformer was distinguished by an arrangement of disulphide bonds [11]–[14]: A and B isoforms have a symmetrical arrangement of Fab subunits while A/B does not. Studied here, the hIgG2 molecule resembles an A/B conformer due to asymmetrical Fab arrangement. Some additional digital experiments in EMAN could be made to ensure our conclusion.

#### Conformers in the sample

We used *multirefine* in EMAN [30] to test for presence of hIgG2 conformers in our dataset. We programmed the *multirefine* routine to create independent models for up to three conformers. It produced only one converged asymmetrical model (data not shown), very similar to the one shown in Figure 3. Changing *multirefine* parameters in different ways did not affect the result. In other words, *multirefine* did not find particles in our hIgG2 dataset suitable to produce other models with other conformation. This indicates that only one conformer was available for 3D reconstruction and it was A/B conformer.

#### hIgG2 symmetry

It is a common belief that most IgG molecules are symmetrical. This dogma travels from one textbook to another along with the idea that the IgG molecule is also flat (represented by “Y” or “T”). In reality, only a few incomplete structures deficient in hinge are flat and symmetrical [7], [36]–[38]. The rest of existing structures, in fact, are not symmetrical and are not flat [6], [8], [20], [27]. The hIgG2 presents a special case where disulphide bonds between Fab and Fc are arranged in two different ways creating three isoforms or conformers, of which A and B are symmetrical, while A/B is asymmetrical. We used digital experiments in EMAN [30] to evaluate how symmetry implementation might affect the resulting hIgG2 model. When we implemented C2 symmetry in the 3D reconstruction routine, the model did not converge. In other words, EMAN was not able to create a trustworthy model with the parameters implemented. When C2 symmetry was released in the reconstruction routine, the model successfully converged. The model converged even when symmetry was released in the middle of calculations. The opposite was true as well; implementation of C2 symmetry in the middle of calculations caused the model to stop converging. We also performed similar experiments in *multirefine* with identical results. Based on this, we conclude that only an asymmetrical model (A/B-type conformer) is in agreement with our data.

These digital experiments confirmed that our asymmetrical model is the single solution for the existing dataset. Thus, the model has been identified as an A/B isoform (conformer) of the hIgG2.

### Discussion of hIgG2 model's resolution

The obtained model resolution of 1.78 nm is reasonable for negatively stained samples. We also observed details that may exceed this resolution. We are able to see in segmented volumes the C- and V-parts of Fab; C_H_2 and C_H_3 parts in Fc (see Fig. 4). Most of these structures are similar to known structures, which leads us to believe that the obtained hIgG2 model is real and correctly represents the sample. Nevertheless, we vigorously tested the obtained model (see above) to insure that it properly represented the sample. All tests show that the model was true to the dataset – it was not possible to produce another structure from the same dataset.

A presence of details beyond the stated resolution of 1.78 nm may be explained by the actual resolution being higher than estimated by FSC  = 0.5 criterion. The FSC  = 0.5 criterion is controversial [41]; in modern cryo-EM 3D reconstruction, FSC  = 0.5 criterion is not used and resolution is estimated by the presence of the known details [42].

Further evidence that our model has a better resolution is our observation that X-ray models for Fab and Fc with resolution reduced to 1.8 nm (not shown) do not show details visible in EM hIgG2 model. Experimentally, we established that X-ray models reduced to 1 nm resolution were comparable with our 1.78 nm resolution model (Fig. S2). In our opinion, there are a number of conditions that contributed to the high quality of our model: (a) quality of the sample, its structural and biochemical homogeneity; (b) negative staining technique (see Materials and Methods), which leads to high-quality images with uniform stain distribution that does not obscure fine details; (c) random orientation of the particles on the support film; (d) negative staining dramatically improves the precision of alignment in 3D reconstruction routine because of high signal-to-noise ratio; (e) because of the limited number of particles, most classes contain fewer particles or even a single particle, which prevents details from degrading during the averaging – note the uniform Euler space filling in Figure S1C.

### hIgG2 3D structure and biology of immunoglobulines

hIgG2 3D model is the first-ever model of the whole immunoglobuline obtained by electron microscopy. It shows in detail the structure and relationship between subunits in hIgG2. The resolution of details is so that individual domains are resolved in segmentation maps: VL&VH domains as well as V- and H parts of Fab (Fig. 4A, B). Similarly, C_H_2 and C_H_3 domains have been resolved in Fc (Fig. 4A, C). Such resolution permits direct correlation between numerous indirect structural data and actual 3D structure of the whole intact hIgG2 molecule. For instance, there is a direct correlation between hIgG2 model and hIgG2 isoform, type A/B described by Dillon et al. (2008) [11]. The hIgG2 model opened up the whole area of digital experiments to study the interaction between the model and different receptors etc.

From an applied point of view, there is a growing need for human or humanized monoclonal IgG antibodies for therapy for different diseases [16], [43], [44]. The lower ability of hIgG2 antibodies to bind Fc gamma receptor or activate complement through the classical pathway may be exploited when a therapeutic antibody with less aggressive activities than IgG1 is required, e.g. for inhibition of pro-inflammatory cytokines etc. In fact, therapeutic hIgG2 is already in commercial production [45]–[47].

## Conclusions

The 3D model of the hIgG2 presented here provides direct evidence for an asymmetrical shape of the hIgG2 molecule in which one Fab is in tight contact with the upper portion of Fc (C_H_2 domains) and possible hinge region. The model is in good agreement with the data of DSC and fluorescence measurements, which indicate that two types of Fab subunits, differing in thermostability, are presented in the sample simultaneously. The hIgG2 studied here has been identified as an isoform A/B [11]. The model provides a structural basis for understanding the mechanism of suppression of the effector functions in the hIgG2, isoform A/B: apparently, the upper part of the Fc subunit is corrupted and partially unavailable due to tight interaction with Fab subunit(s).

Furthermore, 3D reconstruction in combination with negative staining provides valuable information regarding the 3D structure of the hIgG2. The 3D reconstruction approach described in this paper is quick, robust and does not require special equipment. It may be used in many fields for quick structural analysis.

## Materials and Methods

### Preparation of purified hIgG2

The hIgG2 was purified from the plasma of a patient coded as a Mat with therapeutic plasma exchange involving extra-corporeal processing of a patient's blood to remove paraprotein – monoclonal immunoglobulin. The procedure was done in the Institute of Hematology and Intensive Care, Moscow in accordance with the USSR Ministry of Public Health instruction for handling human samples. The hIgG2 was purified to >95% homogeneity by precipitation with (NH_4_)_2_SO_4_, ion-exchange chromatography using DEAE-Sephadex followed by gel filtration with AcA-34 [21]. The sample was stored at 4°C up to one month before analysis. Before usage, the sample was subjected to gel filtration with Ultragel ACA-34 to remove degradation products and aggregates and re-tested for quality assurance.

### Thermodynamic and fluorescent studies

DSC experiments were performed using a computerized DASM-4A microcalorimeter [48]. The protein concentration was in the range of 1.5–4.0 mg/ml. The partial heat capacity of the protein was calculated from the calorimetric data as described previously [48]. The partial specific volume was 0.73 ml/g. For fluorescence studies of hIgG2, C_H_1 and/or C_H_2 domains were selectively labeled with FITC in accordance with the procedure described in [22], [29]. Fluorescence measurements were performed on a fluorescence spectrophotometer MPF-44A (Perkin Elmer, USA) at protein concentration 0.2–1.0 mg/ml. Fluorescence intensity was measured in diapason from 460 to 600 nm. The wavelength of inducing light was 450 nm. All samples were equilibrated in 20 mM phosphate buffer, pH 6.0 before measurements.

### Electron Microscopy

For EM analysis, only samples of hIgG2 stored for 20 days at 4°C, were available. Just before EM experiments, the sample was purified using gel-filtration with Ultragel ACA-34 equilibrated with 20 mM ammonium acetate, pH 7.8. Samples did not show any signs of degradation or aggregation. For EM, the concentration was adjusted to A_280_  = 0.01. All samples were in 20 mM ammonium acetate, pH 7.8 and at 4°C. Negative staining was carried out using the approach described by Valentine [49] with modification as described below. The atomically flat surface of mica was used to produce a carbon support film. Ultrathin carbon film was evaporated on top of the mica using a homemade electron gun [50]. The thickness of the carbon film, measured by a quartz-oscillator thickness monitor, was 1.4 nm. A small piece of mica with carbon on top was slowly inserted into a 0.25–0.5 ml drop with the sample at a 45° angle, so that the carbon floated on the drop but was still attached to the mica. After 1 minute adsorption, mica was carefully lifted up with carbon still attached and inserted into the drop with 1% aqueous uranyl acetate staining solution for 1 minute in the exact way it was done the first time. Grids with custom-made carbon holey film were carefully placed (holey film facing the drop) on the floated carbon film. Grids with attached carbon film were lifted up, blotted and air-dried. Grids were analyzed in JEM100C (JEOL, Japan) transmission electron microscope at magnification x60000. EM plates with images were scanned using Nikon Cool SuperScan 9000 scanner at 0.21 nm/pix.

### Three-dimensional reconstruction

Three-dimensional reconstruction was performed using EMAN 1.9 (**E**lectron **M**icrograph **AN**alysis) software [30]. The detailed procedure is described by Ryazantasev et al. [51]. Briefly, digital images were selected in *ctfcon* (EMAN) that were close to focus and were astigmatism-free. Only two plates with approximately 5000 particles satisfied that criterion. In the next step, 3900 individual particles were selected using *boxer* and *autobox* (EMAN) with a box size of 98×98 pixels (0.21 nm/pix). We tried 4 initial models – 5 random blobs of different sizes, ellipsoid with 1∶2 axis ratio, ellipsoid with 1∶3 axis ratio and sphere. In the final 3D reconstruction, Gaussian featureless ellipsoid with a 1∶2 axis ratio was used as an initial model. Reconstruction procedure was considered successful if models converged, FSC curve had a typical shape and Euler angle space filled up relatively uniformly. During the refinement process, the model and its projections were compared to corresponding class-averages and raw particles. Strict criteria were used to differentiate between “bad” and “good” particles; particles significantly different from the model projections were removed from the dataset in the final reconstruction. In addition, a strict parameter in refine routine was used, *classkeep*  = 0.3. To determine whether the dataset was homogeneous, *multirefine* was used (EMAN). To test the symmetry of the model, calculations with C2 symmetry and without symmetry were performed. The resulting model was tested for resolution using Eo-*test* (EMAN). A cut-off of 0.5 in the FSC was used to estimate the model resolution. The model density cut-off was determined in the EMAN refine routine to enclose a volume corresponding to a molecular mass of 160 kDa, assuming a protein density of 1.35 g/cm^3^ [30]. 3D rendering, manipulation and segmentation were performed using Chimera software [35] with segmentation plug-in, the *segger* [39]. For all computing intense calculations, UCLA Hoffman2 Linux cluster was used.

### Accession number

The hIgG2 electron microscopy map has been deposited to EBI with accession no. EMD-912.

## Supporting Information

Figure S1 **hIgG2 3D reconstruction characterization.** (A) Field of the negatively stained with uranyl acetate hIgG2 sample used for 3D reconstruction; insert is a Fourier transform. For this work, the material suitable for 3D reconstruction was limited. We selected EM plates which were close to the focus (see insert). The EM field revealed numerous particles in different orientations. There was no evidence of predominant orientation. The number of particles in the field was quite high, which made particle selection more challenging. We used *autoboxer* in EMAN to select (“box”) the particles. The advantage of using *autoboxer* was that it was less biased and more likely to select random particles without predominant orientation. We collected 3900 particles. Because of the high concentration of particles, sometimes part of another particle could be included in the “box” – to mitigate this we used EMAN's *automask* feature. It essentially creates a small sphere in the center of the “box” which expands until it touches the particle. It further expands to fill up the particle and continues until the background threshold is reached. Then it creates a fussy Gaussian edge. Every single particle in our dataset was checked to ensure that the mask did not obscure the actual particle. (B) Comparison of model projections (odd numbered, left in each pair) and particle images from corresponding classes (even numbered, right in each pair). Note that relatively tight mask was used to mitigate a high density of the particles in the sample. Mask was adjusted so that it did not obscure any details; the whole particle is within the mask border. (C) Euler space filling. Gray and white (brighter area – more particles in the class) indicates the space occupied by classes. Solid black areas indicate missed classes. (D) FSC (Fourier Shell Correlation function) plot indicating 1.78 nm resolution at FSC  = 0.5 obtained by *eotest*, EMAN.
(TIF)

Figure S2 **Identification of the hIgG2 subunits.** (A–B) Fab-1 subunit; (C–D) Fc subunit. The models derived from X-ray crystallography are pictured in gray. Identification of the subunits of the hIgG2 was based on known Fab and Fc shapes from X-ray crystallography [28], [52]. Fab subunit, which was distant from other parts of the hIgG2 molecule was identified first by its typical appearance; it is called Fab-1. EM Fab-1 model was compared to the Fab subunit obtained from X-ray crystallography (pictured in gray). For better compatibility with EM model, X-ray crystallography model's resolution was reduced. Initially, we reduced the resolution to 1.8 nm, but the result was unsatisfying; the X-ray model lost most of its features and comparison to the EM model was not possible. Therefore, the resolution that could be comparable to the EM model was established through experimentation at 1 nm. Fab-1 has an overall shape similar to that found in the X-ray structure. The V- and C-parts of Fab-1 are very similar to the X-ray structure. However, the gap between V- and C-parts of EM Fab-1 is bigger than in the X-ray model. (C–D) shows the structure of the hIgG2 Fc subunit in comparison to the model of the Fc subunit derived from X-ray crystallography. Note that the X-ray model was filtered down to 1 nm resolution for compatibility with EM data as for Fab. The Fc subunit has a teardrop shape, which is different from the X-ray model. The teardrop shape of the Fc subunit in negatively stained samples of other IgGs was previously observed (see text of the article for details). The gap between C_H_2 and C_H_3 portions of the Fc subunit on the model is less pronounced than in the corresponding X-ray structure. The arrangement and shape of C_H_3 domains appears similar to the X-ray structure, while that of C_H_2 domains does not. In the side view, it is noticeable that the plane of C_H_2 domains is tilted substantially to the plane of C_H_3 domains – making the Fc subunit bend along the long axis. Note that this bend is visible only in the side view. The interface between Fab-2 and Fc subunits (magenta in D) is large, indicating tight interaction between two subunits. Models in (A, C) and (B, D) are rotated 90^o^ clockwise along the vertical axis.
(PNG)

Figure S3 **Connection of Fab-1 to Fc fragment.** (A) Stereo image of the area where Fab-1 connects to Fc. (B) Cross section through connector illustrates the continuous density between Fab-1 and Fc subunit. Based on the architecture of the IgG molecule, we assume that the connector bridges the constant part of Fab-1 and upper part of the C_H_2 domain. (C) Box indicating the approximate area captured in (A–B). Note, in (A–B) the density threshold cutoff was adjusted to make the connector visible, making the molecule looks “fatter.” The orientation of the hIgG2 molecule in (C) is slightly different than in (A–B).
(PNG)
