# Supplementary figures and images for: Three-Dimensional Structure of the Human Myeloma IgG2
Source: PLoS One. 2013 Jun 7;8(6):e64076. doi: 10.1371/journal.pone.0064076 (PMC3676413; doi:10.1371/journal.pone.0064076)

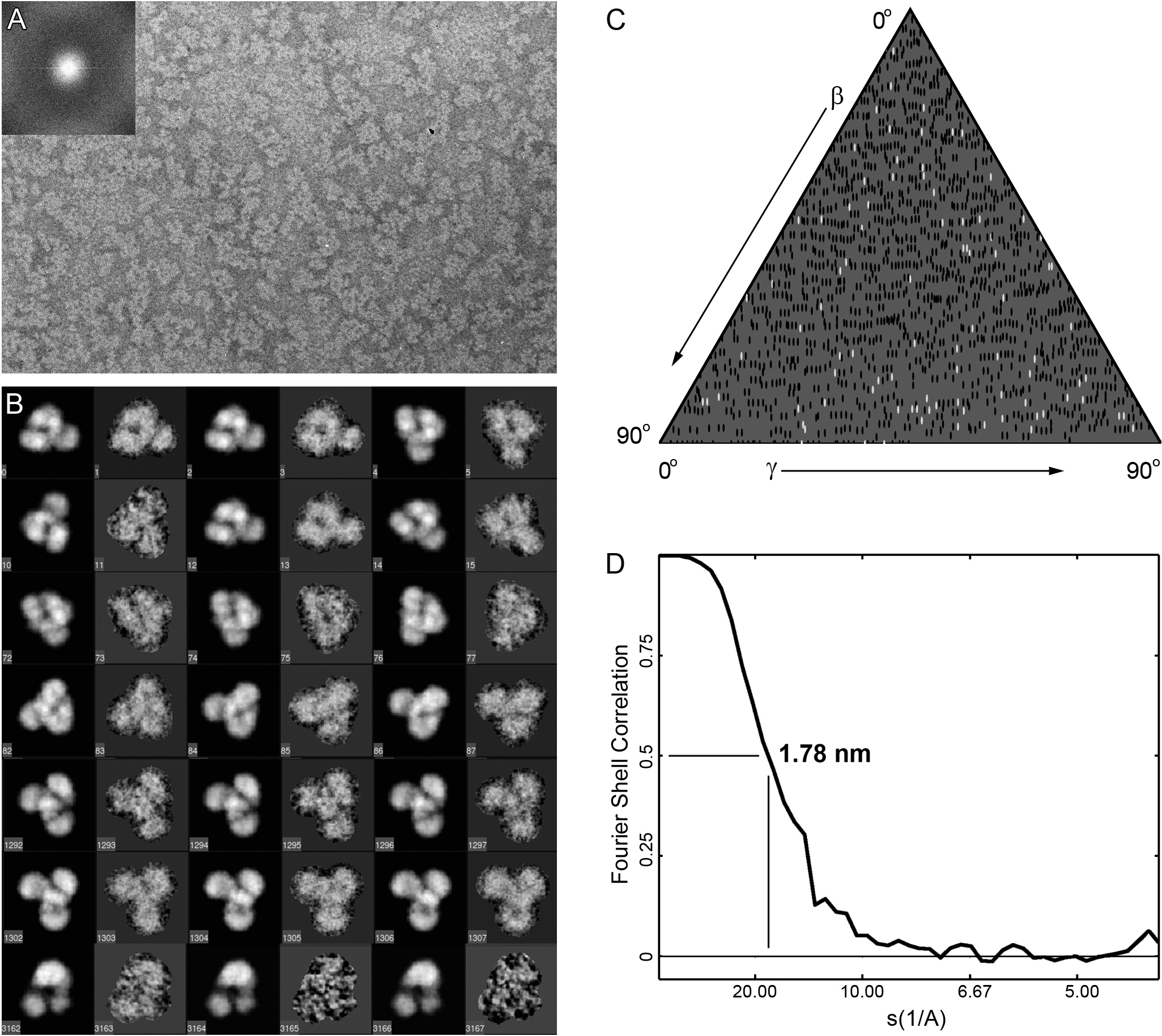

Supplement: Figure S1 — hIgG2 3D reconstruction characterization. (A) Field of the negatively stained with uranyl acetate hIgG2 sample used for 3D reconstruction; insert is a Fourier transform. For this work, the material suitable for 3D reconstruction was limited. We selected EM plates which were close to the focus (see insert). The EM field revealed numerous particles in different orientations. There was no evidence of predominant orientation. The number of particles in the field was quite high, which made particle selection more challenging. We used autoboxer in EMAN to select (“box”) the particles. The advantage of using autoboxer was that it was less biased and more likely to select random particles without predominant orientation. We collected 3900 particles. Because of the high concentration of particles, sometimes part of another particle could be included in the “box” – to mitigate this we used EMAN's automask feature. It essentially creates a small sphere in the center of the “box” which expands until it touches the particle. It further expands to fill up the particle and continues until the background threshold is reached. Then it creates a fussy Gaussian edge. Every single particle in our dataset was checked to ensure that the mask did not obscure the actual particle. (B) Comparison of model projections (odd numbered, left in each pair) and particle images from corresponding classes (even numbered, right in each pair). Note that relatively tight mask was used to mitigate a high density of the particles in the sample. Mask was adjusted so that it did not obscure any details; the whole particle is within the mask border. (C) Euler space filling. Gray and white (brighter area – more particles in the class) indicates the space occupied by classes. Solid black areas indicate missed classes. (D) FSC (Fourier Shell Correlation function) plot indicating 1.78 nm resolution at FSC = 0.5 obtained by eotest, EMAN. (TIF) [file pone.0064076.s001.tif]

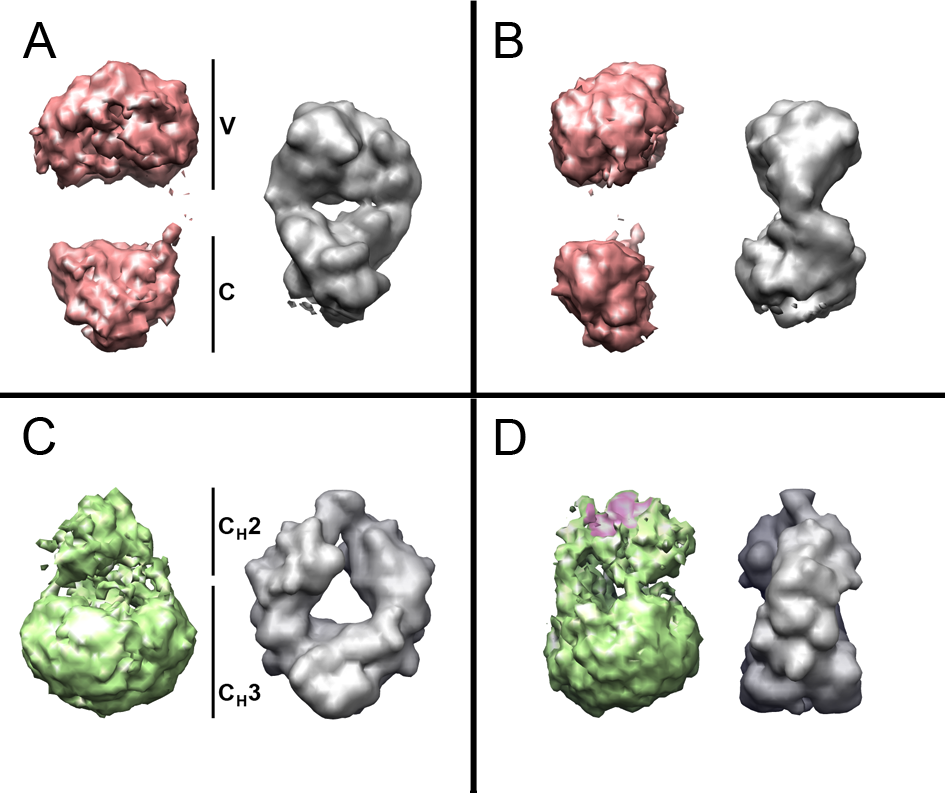

Supplement: Figure S2 — Identification of the hIgG2 subunits. (A–B) Fab-1 subunit; (C–D) Fc subunit. The models derived from X-ray crystallography are pictured in gray. Identification of the subunits of the hIgG2 was based on known Fab and Fc shapes from X-ray crystallography [28], [52]. Fab subunit, which was distant from other parts of the hIgG2 molecule was identified first by its typical appearance; it is called Fab-1. EM Fab-1 model was compared to the Fab subunit obtained from X-ray crystallography (pictured in gray). For better compatibility with EM model, X-ray crystallography model's resolution was reduced. Initially, we reduced the resolution to 1.8 nm, but the result was unsatisfying; the X-ray model lost most of its features and comparison to the EM model was not possible. Therefore, the resolution that could be comparable to the EM model was established through experimentation at 1 nm. Fab-1 has an overall shape similar to that found in the X-ray structure. The V- and C-parts of Fab-1 are very similar to the X-ray structure. However, the gap between V- and C-parts of EM Fab-1 is bigger than in the X-ray model. (C–D) shows the structure of the hIgG2 Fc subunit in comparison to the model of the Fc subunit derived from X-ray crystallography. Note that the X-ray model was filtered down to 1 nm resolution for compatibility with EM data as for Fab. The Fc subunit has a teardrop shape, which is different from the X-ray model. The teardrop shape of the Fc subunit in negatively stained samples of other IgGs was previously observed (see text of the article for details). The gap between CH2 and CH3 portions of the Fc subunit on the model is less pronounced than in the corresponding X-ray structure. The arrangement and shape of CH3 domains appears similar to the X-ray structure, while that of CH2 domains does not. In the side view, it is noticeable that the plane of CH2 domains is tilted substantially to the plane of CH3 domains – making the Fc subunit bend along the long axis. Note that [file pone.0064076.s002.png]

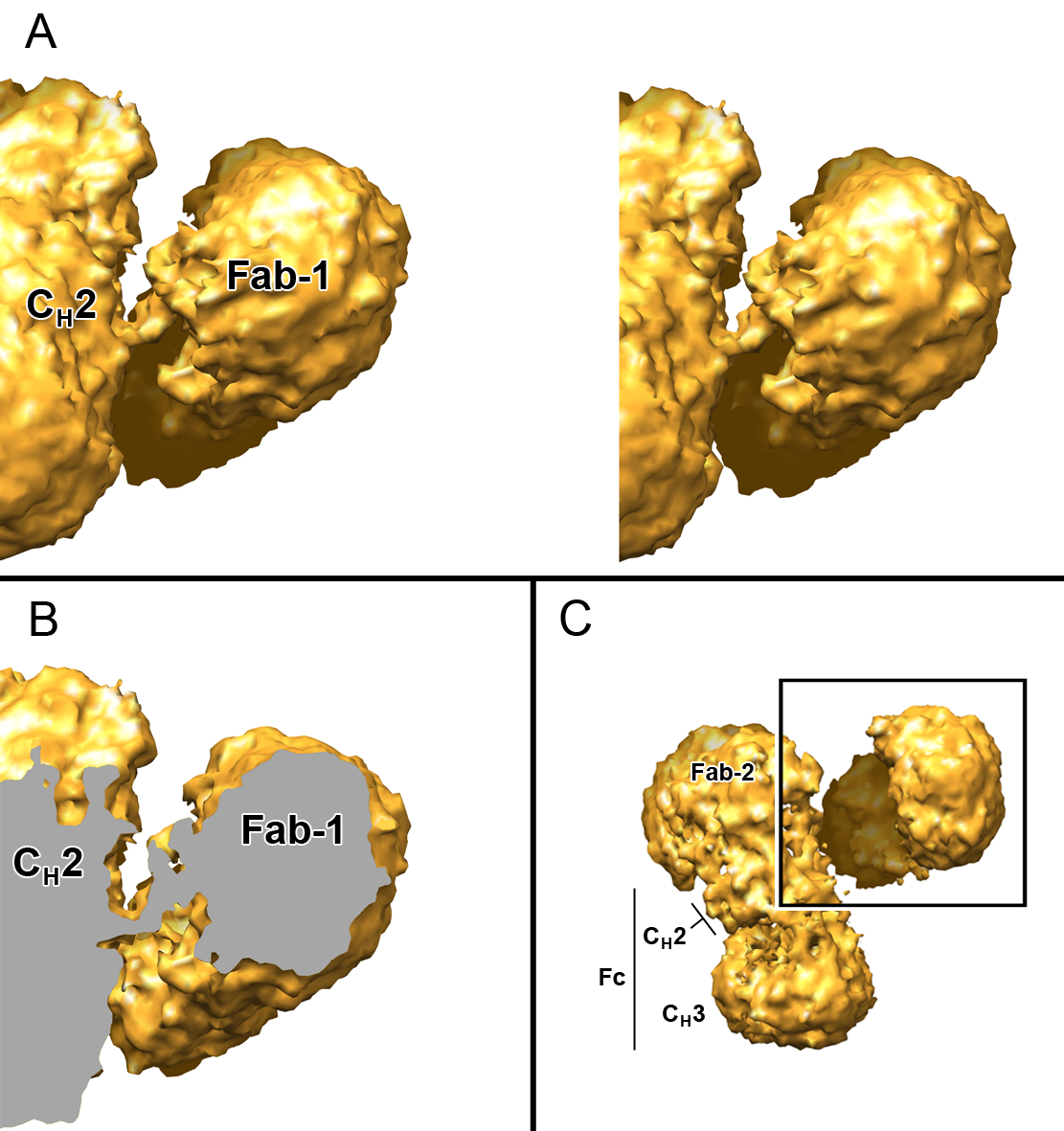

Supplement: Figure S3 — Connection of Fab-1 to Fc fragment. (A) Stereo image of the area where Fab-1 connects to Fc. (B) Cross section through connector illustrates the continuous density between Fab-1 and Fc subunit. Based on the architecture of the IgG molecule, we assume that the connector bridges the constant part of Fab-1 and upper part of the CH2 domain. (C) Box indicating the approximate area captured in (A–B). Note, in (A–B) the density threshold cutoff was adjusted to make the connector visible, making the molecule looks “fatter.” The orientation of the hIgG2 molecule in (C) is slightly different than in (A–B). (PNG) [file pone.0064076.s003.png]
